# Acromelic frontonasal dysostosis and ZSWIM6 mutation: phenotypic spectrum and mosaicism

**DOI:** 10.1111/cge.12721

**Published:** 2016-02-03

**Authors:** S.R.F. Twigg, L.B. Ousager, K.A. Miller, Y. Zhou, S.C. Elalaoui, A. Sefiani, G.S Bak, H. Hove, L.K. Hansen, C.R. Fagerberg, M. Tajir, A.O.M. Wilkie

**Affiliations:** ^1^ Clinical Genetics GroupWeatherall Institute of Molecular Medicine, University of OxfordOxfordUK; ^2^ Department of Clinical GeneticsOdense University HospitalOdenseDenmark; ^3^ Human Genomics CenterFaculty of Medicine and Pharmacy of RabatRabatMorocco; ^4^ Department of Medical GeneticsNational Institute of HealthRabatMorocco; ^5^ Department of Obstetrics and GynecologyOdense University HospitalOdenseDenmark; ^6^ Department of Clinical GeneticsCopenhagen University Hospital RigshospitaletCopenhagenDenmark; ^7^ Department of PaediatricsHans Christian Andersen Children's Hospital, Odense University HospitalOdenseDenmark

**Keywords:** frontonasal malformation, mosaicism, preaxial polydactyly, ZSWIM6

## Abstract

Acromelic frontonasal dysostosis (AFND) is a distinctive and rare frontonasal malformation that presents in combination with brain and limb abnormalities. A single recurrent heterozygous missense substitution in ZSWIM6, encoding a protein of unknown function, was previously shown to underlie this disorder in four unrelated cases. Here we describe four additional individuals from three families, comprising two sporadic subjects (one of whom had no limb malformation) and a mildly affected female with a severely affected son. In the latter family we demonstrate parental mosaicism through deep sequencing of DNA isolated from a variety of tissues, which each contain different levels of mutation. This has important implications for genetic counselling.

Acromelic frontonasal dysotosis (AFND; MIM 603671) is characterized by a combination of characteristic frontonasal malformation (FNM) with limb defects and anomalies of the brain and usually occurs as a sporadic disorder. Following initial description in a review of the diverse presentations of FNM 1, Verloes et al. proposed AFND as a distinct entity 2; subsequent reports have highlighted characteristic features of severe hypertelorism, ptosis, median cleft face with distinctive nasal bifurcation and widely separated nasal alae, parietal foramina, variable brain abnormalities including dysgenesis of the corpus callosum, hydrocephalus and interhemispheric lipoma, limb anomalies with preaxial polydactyly of the feet, tibial aplasia or hypoplasia, and talipes equinovarus 3, 4, 5. Although mainly arising sporadically, possible vertical transmission 5 suggested a dominant mechanism, subsequently confirmed by identification of the underlying heterozygous mutation in four AFND cases 6. These four cases were found to carry an identical mutation of *ZSWIM6* (MIM: 615951; c.3487C>T; p.Arg1163Trp), all apparently *de novo* in origin. In one subject, a reduced ratio of mutant to wild‐type allele indicated that the mutation was present in mosaic state and had likely arisen post‐zygotically; in another family, mild phenotypic features in the father were speculated to be caused by mosaicism, although no evidence of *ZSWIM6* mutation was found in the blood from this individual 6.

We describe four additional cases of AFND carrying the *ZSWIM6* c.3487C>T variant. Limb anomalies, usually part of the AFND phenotype, were absent in one case with a constitutive *de novo* mutation, establishing that this is not an absolute requirement for diagnosis. In a mildly affected parent we demonstrate mosaicism, confirming that this mechanism can result in a milder phenotype within the FNM spectrum.

## Materials and methods

### Subjects

The study was approved by Oxfordshire Research Ethics Committee B (reference C02.143) and Riverside Research Ethics Committee (reference 09/H0706/20); written informed consent was obtained from all participants by the referring clinicians. Karyotyping of all subjects was normal; although array comparative hybridisation (aCGH; Agilent 244 K) in Subjects 1‐1 and 1‐2 showed a 3.2 Mb duplication of 16p12.3‐p13.1 that had arisen *de novo* in the mother, this appears to be coincidental to the AFND phenotype. DNA was extracted from peripheral blood samples (Subjects 1‐1, 1‐2, 2 and parents of Subjects 2 and 3), an aminiocentesis sample (Subject 3), and buccal brushings, saliva, urine and skin (Subject 1‐1). The resequencing panel consisted of 27 individuals with mild to severe FNMs, with or without extracranial abnormalities, and lacking a molecular diagnosis.

### Molecular analysis

A 370 bp fragment covering the *ZSWIM6* (Refseq NT_034772.7) exon 14 c.3487C>T variant was amplified using primers E14F 5′‐GCTATAATACCTCTGGTGGTCAAGAGTG‐3′ and E14R 5′‐CCCGAACCAACATCATCAGTTTC‐3′. Amplification was carried with 0.5 U of FastStart polymerase (Roche Diagnostics, Burgess Hill, UK) in a total volume of 20 µl containing 15 mM Tris–HCl (pH 8.0), 50 mM KCl, 2.5 mM MgCl_2_, 100 μM each dNTP and 0.4 μM primers. Cycling conditions consisted of an 8 min denaturation step at 94°C, followed by 33 cycles of 94°C for 30 s, 63°C for 30 s and 72°C for 30 s, with a final extension at 72°C for 10 min. This product was sequenced using BigDye Terminator v3.1 (Applied Biosystems, Foster City, CA, USA). Deep sequencing on the Ion Torrent PGM platform was used to quantify the proportions of wild‐type to mutant allele in genomic DNA. Fragments of 220 bp spanning the c.3487C>T variant were generated (*ZSWIM6*‐specific primers: Exon14F 5′‐GCCTACATCAACACAACGCACTCACGG‐3′ and Exon14R 5′‐CATACAAGATCTATCAACCAAACCTCTCCC‐3′ with a 10 bp barcode incorporated into either the reverse or forward oligonucleotide and flanked by Ion Torrent P1 and A adapter sequences). The P1 and A adapter sequences were flipped so that Ion Torrent sequencing could be carried out in both directions. The high‐fidelity Taq polymerase Q5 (NEB, Hitchin, UK) was used for amplification (0.02 U/µl) in a reaction volume of 25 µl containing 0.5 μM primers, 25 mM Tap‐HCl (pH 9.3), 50 mM KCl, 2 mM MgCl_2_, 1 mM β‐mercaptoethanol and 200 μM each dNTP. Cycling was carried out as described above except the cycle number was reduced to 30 and the annealing temperature was 60°C. Amplification products were purified with AMPure beads (Beckman Coulter, High Wycombe, UK) and emulsion polymerase chain reaction (PCR) and enrichment performed with the Ion PGM Template OT2 200 Kit (Life Technologies) according to the manufacturer's instructions. Sequencing of enriched templates was performed on the Ion Torrent PGM (Life technologies, Carlsbad, CA, USA) for 125 cycles using the Ion PGM Sequencing 200 kit v2 on an Ion 316 chip. Data were processed with Ion Torrent platform‐specific pipeline software v4.2.1. As the variant must be present at a level of 50% in a heterozygous individual, and at 0% in a normal control, the forward and reverse deep sequencing read counts were separately normalized using the data from Subject 1‐2 and a control, and the average of the two corrected percentages calculated.

## Results

### Clinical description

**Figure 1:**
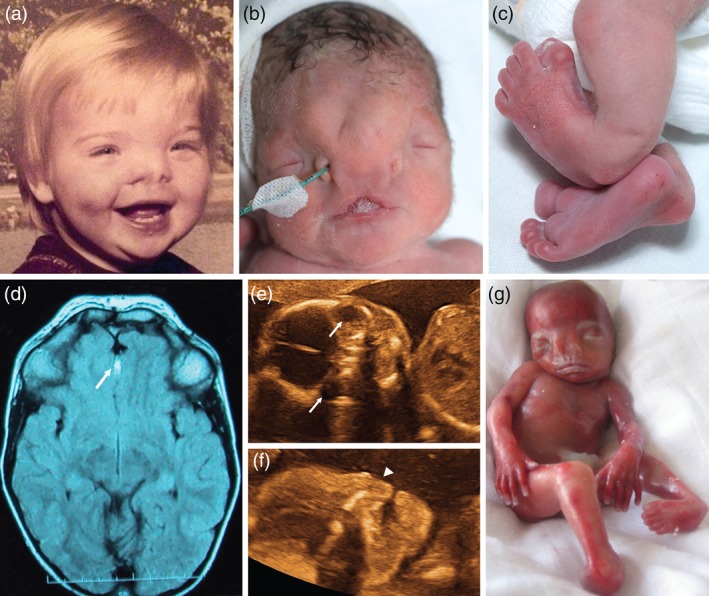
Clinical features of individuals with ZSWIM6 c.3487C>T mutations. (**a**) Subject 1‐1 showing facial features at about 1 year of age. Note hypertelorism and bifid nasal tip. (**b**,**c**) Neonatal appearance of Subject 1‐2, with severe FNM, hypertelorism, carp‐shaped mouth with notch in upper lip (**b**), and bifid great toes and clubfoot (**c**). (**d**) Brain magnetic resonance imaging of Subject 2 showing interhemispheric lipoma (white arrow), and severe hypertelorism. (**e**,**f**) Ultrasound images of Subject 3 showing orbital hypertelorism (**e**, arrows indicate the eyes) and hypoplastic nose (**f**, arrowhead). (**g**) Clinical appearance of Subject 3 with FNM, hypertelorism and clubfoot. Polydactyly is absent.

The clinical features of Subjects 1‐1, 1‐2, 2 and 3 are summarized in Table 1 and shown in Figure 1. Subject 1‐2 was born at 32 weeks' gestation (birth weight: 1580 g) and diagnosed with AFND due to the association of severe FNM and limb abnormalities. Currently aged 7 years, he has severe neurocognitive and motor delay and is unable to walk and communicate with words. His mother, Subject 1‐1, who had undergone numerous surgical procedures to reshape the frontonasal region, was suspected to have a milder form of the same disorder. She has hypertelorism with a short, broad nose and bifid nasal tip, but normal intelligence and no extracranial features. Subject 2, the third of four children born to unrelated and unaffected parents, is a 12‐year‐old boy with severe psychomotor delay who was not diagnosed with AFND due to absence of limb abnormalities. Extracranially he had scoliosis, cryptorchidism and micropenis. Subject 3 was a female fetus with abnormalities detected by ultrasound at 19 + 3 weeks' gestation, including facial malformation with hypertelorism and broad glabella, nasal hypoplasia and bilateral talipes equinovarus. Following elective termination of pregnancy, postnatal examination revealed features in keeping with a diagnosis of AFND, including median facial cleft and bilateral tibial hypoplasia, although polydactyly was absent. No other family members had similar abnormalities.

**Table 1:** Clinical features of subjects with ZSWIM6 c.3487C>T; p.Arg1163Trp

Subject #	Gender^a^	Craniofacial				Brain		Limbs	Other
		Eyes	Nose	Mouth	Skull	Morphology	Development		
1‐1^b^	F	Hypertelorism	Wide nasal bridge, short nasal ridge, bifid nasal tip	Normal	Normal	Normal	Normal	Normal	—
1‐2	M	Severe hypertelorism, downslanting palpebral fissures	Wide nasal bridge, widely spaced nasal alae, widely separated slit‐like nares	Carp‐shaped mouth, midline notch in upper lip, cleft palate	Bony defect of anterior cranial fossa, parietal foramina	Interhemispheric lipoma, partial agenesis of the corpus callosum	Severe motor and neurocognitive delay	Normal upper limbs, bilateral tibial hemimelia, bilateral bifid first toe, bilateral clubfoot	—
2	M	Hypertelorism, bilateral ptosis, downslanting palpebral fissures, bilateral cataract	Wide nasal bridge, short nasal ridge, bifid nasal tip, widely spaced nasal alae, widely separated slit‐like nares	Carp‐shaped mouth, long philtrum, midline notch in upper lip, cleft palate	Bony defect of anterior cranial fossa	Anterior interhemispheric lipoma	Severe psychomotor delay, absence of speech, does not walk aged 8 years	Normal	Micropenis, cryptorchidism, scoliosis
3^c^	F	Hypertelorism, downslanting palpebral fissures	Aplasia/hypoplasia of the nasal bones, wide nasal bridge, bifid nasal tip	Midline notch in upper lip	—	—	—	Normal upper limbs, bilateral tibial hypoplasia, bilateral club foot	—

a F, female, M, male.

b Mosaic for the mutation.

c Pregnancy terminated at 20 weeks' gestation.

### Molecular analysis

**Figure 2:**
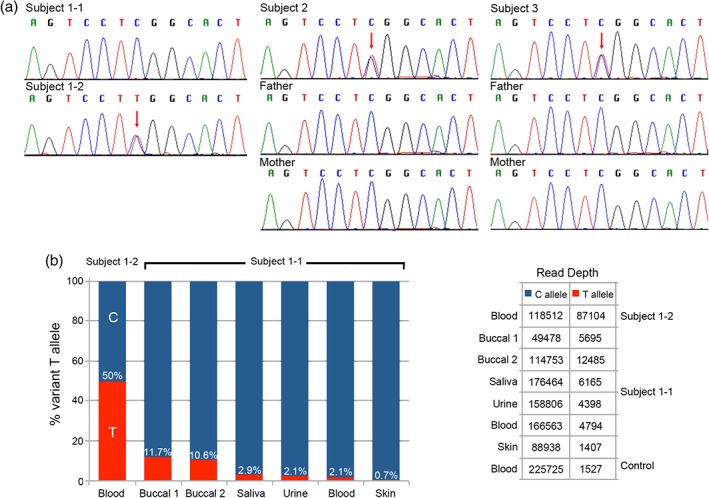
ZSWIM6 sequence analysis. (**a**) Sequence chromatograms showing ZSWIM6 c.3487C>T in Subjects 1‐2, 2 and 3 (red arrows). The C>T variant is absent in Subject 1‐1 (DNA from peripheral blood) and the parents of Subjects 2 and 3. (**b**) Deep sequence analysis for ZSWIM6 c.3487C>T. The left hand panel shows the percentage of the variant T allele detected in Subject 1‐1 and 1‐2. The value for Subject 1‐2 has been corrected to 50% and all other figures adjusted accordingly. The T allele is shown in red and the C allele in blue. The right hand panel shows the uncorrected read depths achieved for each sample.

Screening for *ZSWIM6* c.3487C>T in a cohort of 27 FNM cases revealed the presence of this variant in three individuals, Subjects 1‐2, 2 and 3 (Fig. 2a). The ratio of mutant to wild‐type allele was approximately 50:50 in each case. As the variant was not detected in blood from Subject 1‐1, the mildly affected mother of Subject 1‐2 and in whom mosaicism was suspected, we analyzed DNA from four other tissues (buccal brushings, saliva, skin and urine). Sanger sequencing was inconclusive although a subtle drop in peak height of the wild‐type allele was evident in the two buccal samples, suggesting the presence of the variant allele (data not shown). This prompted us to undertake more sensitive Ion Torrent‐based deep sequencing, which identified the mutant variant in all five tissue samples from Subject 1‐1 (Fig. 2b). The average sequencing depth obtained was >152,800 with a lowest read number of 55,173. The percentage of variant allele was highest at ∼11% in the buccal scrapings (equivalent to ∼22% mutant cells), at 3% in saliva, around 2% in urine and blood and lowest at under 1% in skin. The sensitivity of mutation detection using Sanger sequencing is around 6% 7 providing an explanation for why testing of DNA from peripheral blood of Subject 1‐1 was negative.

## Discussion

AFND is an extremely rare FNM with fewer than 20 recognizable cases described in the literature 1, 2, 4, 5, 6, 8, 9, 10. The most consistent clinical features are FNM accompanied by preaxial polydactyly of the lower limbs. The nasal deformity is usually severe, with symmetrical clefting and widely separated slit‐likes nares, while limb anomalies can also include tibial hypoplasia and clubfoot. Recently, a recurrent mutation of *ZSWIM6*, c.3487C>T encoding p.Arg1163Trp, was identified in four AFND individuals 6. ZSWIM6 is a member of a group of proteins, found in bacteria, archaea and eukaryotes, that all contain a SWIM Zn‐finger‐like domain that could function both as a DNA binding domain or in protein–protein interaction 11. Very little is known about the role of ZSWIM6, although the missense substitution identified in AFND is likely to disrupt the function of a highly conserved sin3‐like domain at the C‐terminus of the protein 6. Expression appears to be ubiquitous although higher in the brain, and analysis of AFND patient cells suggests an effect on hedgehog signaling 6. A molecular‐developmental explanation for the specific pattern of malformations occurring in AFND is currently lacking.

In this report we screened a phenotypically diverse FNM cohort for this variant and identified three positive individuals, all of whom shared the characteristic nose with symmetrical, widely separate nostril openings and severe hypertelorism (Fig. 1b,e). This included a previously undiagnosed patient with severe FNM but normal limbs. A confident diagnosis of AFND with normal limbs has only been possible in one previous case, one of the two half‐sisters reported by Warkany et al. 10, where a diagnosis could be made because of the classically affected relative. Our findings imply that similar cases with isolated severe symmetrical FNM should undergo *ZSWIM6* screening. Interestingly, although Subject 3 had lower limb abnormalities, polydactyly was absent, highlighting that this feature may not always be present either.

Although mosaicism had been suspected in the mildly affected parent of a classical AFND patient 6, it was not molecularly confirmed. We prove, through next generation deep sequencing of DNA from multiple tissues, that mosaicism can occur in the mildly affected parents of AFND cases. Notably, the low level of mosaicism found could not be convincingly detected by Sanger sequencing, even of multiple tissues. The use of PCR‐based or capture techniques combined with next generation deep sequencing is an effective method to identify low frequency mosaic mutations that are missed by conventional techniques 12, 13. In our analysis deep sequencing allowed the convincing detection of mutations at less than a 2% level. The finding of mosaicism has important counselling implications for AFND families and the possibility of mosaicism in one of the parents of a child with a germline mutation, whether they are mildly affected or appear normal, should be considered. The phenotype of Subject 1‐1 shows similarities to frontorhiny, a distinct FNM caused by biallelic mutations of *ALX3* 14. We propose that for patients thought to have frontorhiny, but with a negative *ALX3* mutation screen, the possibility of low‐level mosaicism for the *ZSWIM6* mutation should be sought by deep sequencing of multiple tissues.
